# Prevalence and factors associated with stroke risk factors in an urban community of Parakou, Northern Benin, 2016

**DOI:** 10.1371/journal.pgph.0000667

**Published:** 2022-07-01

**Authors:** Yessito Corine Nadège Houehanou, Mendinatou Agbetou, Oyéné Kossi, Maurice Agonnoudé, Hospice Hountada, Thierry Adoukonou

**Affiliations:** 1 National School of Public Health and Epidemiological Surveillance (ENATSE), University of Parakou, Parakou, Benin; 2 Department of Neurology, Borgou-Alibori Departmental University Hospital, Parakou, Benin; African Population and Health Research Center, KENYA

## Abstract

Sub-Saharan Africa faces a heavy burden of stroke due to the growth of its risk factors. We aimed to estimate the prevalence of stroke risk factors and identify the factors associated with metabolic risk factors in the district of Titirou, in Parakou (northern Benin) in 2016. A cross-sectional study was conducted. It included people aged at least 15 years, living in Titirou for at least 6 months, and who had given their written consent to participate in the study. A door-to-door survey was performed from 15 march to 15 July 2016 in each neighborhood until the pre-determined number was reached. Sociodemographic data, medical histories, anthropometric and blood pressure measures were recorded using the WHO STEPS approach. The prevalence of stroke risk factors was calculated, and a multivariable logistic regression was done to identify the factors associated with metabolic risk factors for stroke. A total of 4671 participants were included with a mean age of 27.7±12.9 years and a sex ratio of 0.98. Concerning the behavioral risk factors for stroke, 17.2% were alcohol consumers, 3.5% were smokers, 21.5% had low fruit and vegetable intake, and 51.1% had low physical activity practice. The prevalence of metabolic risk factors for stroke was respectively of 8.7% for obesity, 7.1% for high blood pressure, 1.7% for self-reported diabetes, and 2.2% for dyslipidemia. Age (p<0.001), sex (p<0.001), marital status (p<0.001) and professional occupation (p = 0.010) were associated with obesity. Age was also associated with high blood pressure (p<0.001) and diabetes (p<0.001). Dyslipidemia varied according to smoking (p = 0.033) and low physical activity practice (p = 0.003). The study revealed a significant prevalence of some stroke risk factors. Targeted local interventions for primary prevention of stroke should be promoted in this community.

## Background

Cardiovascular diseases are a public health problem in the world. In 2016, the World Health Organization (WHO) estimated that around 17.9 million people died of cardiovascular disease, meaning one-third of global mortality [1]. Most of these deaths were due to vascular diseases such as heart attack and stroke and occurred in low-and middle-income countries. A study published in 2019 showed that while the incidence and mortality of stroke are declining in high-income countries, they are increasing in low-income countries [2].

Progresses made in the control of infectious diseases in Sub-Saharan Africa (SSA) are threatened by the burden of non-communicable diseases. In fact, many cross-sectional studies realized in Benin between 2012 and 2020 showed a prevalence of stroke that varies from 0.2% to 1.5% [3–5]. Moreover, a study conducted in Parakou from 2012 to 2018 found a 5-year stroke mortality among stroke survivors patients of 23.5% [6]. The cost of stroke treatment is quite expensive. In 2011, the direct hospital cost of stroke treatment in Parakou University hospital was estimated at around 620 USD [7].

Despite the heavy burden of stroke, it can be reduced by carrying out interventions on risk factors. In fact, the promotion of a healthy diet and regular physical activity, smoking cessation, and reducing alcohol consumption are the keys to the primary prevention by controlling metabolic risk factors such as: obesity, high blood pressure, raised blood sugar, and raised blood cholesterol [1].

The changes in food habits and growing sedentary lifestyles are leading to rising frequencies of stroke risk factors in SSA proved by results of STEPS surveys among adults [8]. In Benin, the STEPS survey conducted in 2015 among adults aged of 18 to 69 years old showed that 5% of adults used tobacco and 26.5% of them consumed alcohol. Moreover, about 93.1% of the participants had insufficient fruit and vegetable intake and 15.9% had low physical activity practice with almost a tenth (7.4%) that had obesity, 25.9% high blood pressure, 12.4% raised blood sugar and 4.4% raised blood cholesterol [9]. The survey also showed disparities between regions, areas of residence (rural or urban), and social categories, highlighting the value of comprehensive studies on targeted subpopulations.

For an effective fight against stroke in SSA countries and particularly in Benin, it is necessary to strengthen the primary prevention measures accessible to the local health system and communities. Considering epidemiological data and periodically update them are important for the implementation of targeted interventions.

## Materials and methods

### Aim, design, and frame

We aimed to estimate the prevalence of stroke risk factors and identify factors associated with metabolic risk factors in the district of Titirou, in Parakou (Benin) in 2016. A cross-sectional study was conducted in Titirou, the second most populated district of Parakou. According to the 2013 census, its population was estimated to 25,530 with 12,816 people aged at least 15 years [10]. It is subdivided into seven neighborhoods and is located at 1 km far from the University Hospital.

### Participants and sampling

The study included people aged at least 15 years, resident in Titirou and present at the date of the survey, and who had given their written consent to participate. Those unable to answer questions or those who were missed over three visits were not included.

A door-to-door survey was conducted. In each neighborhood, the researchers randomly determined a direction from the center and randomly chose one side in the selected direction. The houses were visited based on a close-to-close approach. All persons living in households and who met the inclusion criteria were interviewed until the expected number was reached. If in a neighborhood this number was not reached, the investigators returned to the center and the operation was repeated in another direction until the number was finally got.

The sample size was initially calculated for a survey focused on the stroke prevalence with an expected value of 4.6 per 1000, accuracy of 0.002 and an alpha risk of 0.05, giving a minimal number of subjects of 4600. The size by neighborhood was proportional to the number of residents aged at least 15 years.

For our study, this sample size corresponded to an accuracy of 1.5% for the estimation of the prevalence of stroke risk factors, considering a theoretical value of 50%, and an alpha risk of 0.05.

### Variables

Metabolic risk factors for stroke (“yes” or “no”) were the dependent variables. They were defined according to WHO STEPS surveys manual [11]. (1) Obesity was defined by a body mass index (BMI) ≥30 kg/m^2^ that was calculated by dividing the weight (kg) by the square of the size (m); (2) high blood pressure by a systolic blood pressure ≥140 mm Hg and/or a diastolic blood pressure ≥ 90 mm Hg during the survey; (3) diabetes by self-report history of diabetes (at least two results of fast venous blood glucose tests ≥ 1,26 g/l or using diabetes drugs during the survey); (4) dyslipidemia by self-report of history raised total blood cholesterol (at least one result ≥ 2,40 g/l or using statins during the survey).

Sociodemographic and behavioral risk factors for stroke were the independent variables. Behavioral risk factors for stroke (“yes” or “no”) were defined based on the self-report information [11]. (1) “Low fruit and vegetable intake” was defined by the consumption of less than 5 servings of fruit and vegetable per day during the last 12 months; (2) “current smoking” by the consumption of tobacco during the last 12 months; (3) “alcohol consumption” by the consumption of alcoholic beverages during the 30 last days; (4) “low physical activity practice” by the practice of less than 150 minutes per / week of moderate physical activity or the equivalent of vigorous physical activity or a combination of moderate and vigorous activities, during the last 12 months.

### Data collection

Two medical students at the end of their training, helped by 8 students in public health school at the University of Parakou, collected data from 15 March to 15 July 2016. They were trained on data collection tools and supervised by 2 physicians. The data collection form was written in French from the WHO STEPS instrument [11]. It comprised data on sociodemographic factors (age, sex, marital status, occupations, religion, and ethnicity), medical histories (family history of stroke, hypertension, and diabetes), behavioural risk factors (smoking, fruit and vegetable intake, physical activity practice, alcohol consumption), height, weight, and blood pressure. The data collection tools were tested before the survey in another district (“Bannikani”) in Parakou.

At the participant’s home, an individual structured interview was performed in French or the local language. Then, the weight was taken using a weight scale (SECA, United Kingdom) with a precision of 100g. The height was measured using a height rod with a precision of 1 cm (SECA, United Kingdom). After a 5-minute rest, blood pressure was measured with an electronic device (OMRON M3, Japon). The measurement was done in a seated position, on the left arm with the hand resting on a support. Three consecutive measures were taken at 3-minute intervals (between two readings). The mean value of the last two measurements was considered [11].

### Statistical analysis

Statistical analyses were performed using Epi-Info version 7.1.3.10. Software (Epi info, CDC Atlanta, USA). The categorical variables were described by using numbers and percentages and the continuous variables, by using mean ± standard deviation. The Chi2 test or exact Fisher test was used to compare percentages between two groups. The prevalence of stroke risk factors was estimated with a 95% confidence interval. The association between metabolic risk factors for stroke, sociodemographic and behavioral variables was explored through a logistic regression. All the variables with a p-value of 0.20 or less were simultaneously introduced in multivariate analysis by using a step-by-step background approach. The crude and adjusted odds ratios (cOR, aOR) and their confidence intervals at 95% were determined. P-value under 0.05 was considered as significant.

### Ethical considerations

The administrative authorization of the local authorities was obtained before the survey. The local ethical committee of biomedical research of the University of Parakou approved the research (Reference: 029/CLERB-UP/P/SP/R/SA). Each participant approved the written consent form before inclusion. In addition, for persons under the age of 18, written consent from a parent or guardian was obtained prior to their inclusion. The data were managed with confidentiality.

## Results

### General characteristic of participants

A total number of 4671 participants were included with a mean age of 27.6 ± 12.9 years and a sex ratio of 0.98. Among the participants, 68.5% were under 30 years old. Almost a fifth of them lived in couples (16.7%) and had no formal education (16.7%); resellers were most represented (20.4%) (Table 1).

**Table 1:** Prevalence of obesity and high blood pressure according to socio-demographic and behavioral characteristics, Parakou 2016.

	N (%)	Obesity			High blood pressure		
		N (%)	Crude OR [95%CI]	p	N (%)	Crude OR [95%CI]	p
**Sample**	4671 (100)	407 (8.7)			332 (7.1)		
**Age (years)**				<0.001			<0.001
15–29	3198 (68.5)	134 (4.2)	1		131 (4.1)	1	
30–44	927 (19.8)	182 (19.6)	5.6 [4.4–7.1]		89 (9.6)	2.5 [1.9–3.1]	
44–59	362 (7.8)	73 (20.2)	5.8 [4.2–7.9]		79 (21.8)	6.5 [4.2–7.9]	
≥60	184 (3.9)	18 (9.8)	2.5 [1.5–4.2]		33 (17.9)	5.1 [1.5–4.2]	
**Sex**				<0.001			0.653
Female	2365 (50.6)	315 (13.3)	1		172 (7.3)	1	
Male	2306 (49.4)	92 (4.0)	0.3 [0.2–0.4]		160 (6.9)	1.0 [0.8–1.2]	
**School education level**				<0.001			<0.001
None	807 (17.3)	135 (16.7)	1		89 (11.0)	1	
Primary	875 (18.7)	132 (15.1)	0.9 [0.7–1.1]		78 (8.9)	0.8 [0.6–1.1]	
Secondary	2581 (55.3)	124 (4.8)	0.3 [0.2–0.3]		129 (5.0)	0.3 [0.3–0.6]	
Universitary	408 (8.7)	16 (3.9)	0.2 [0.1–0.3]		36 (8.8)	0.8 [0.5–1.2]	
**Marital status**				<0.001			<0.001
Couple	2022 (43.3)	337 (16.7)	1		233 (11.5)	1	
Alone	2649 (56.7)	70 (2.6)	0.2 [0.1–0.3]		99 (3.7)	3.4 [2.6–4.3]	
**Professional activity**				<0.001			<0.001
craftsman	1859 (39.8)	31 (1.7)	1		61 (3.3)	1	
Worker/farmer	1320 (28.3)	170 (12.9)	8.7 [5.9–12.9]		112 (8.5)	2.7 [2.0–3.8]	
Resellers	759 (16.3)	155 (20.4)	15.1 [10.2–22.5]		80 (10.5)	3.5 [2.5–4.9]	
No economic activity/others	733 (15.6)	51 (7.0)	4.4 [2.8–6.9]		79 (10.8)	3.6 [2.5–5.0]	
**Alcohol consumption**				0.204			0.292
No	3867 (82.8)	346 (8.9)	1		282 (7.3)	1	
Yes	801 (17.2)	61 (7.6)	0.8 [0.6–1.1]		50 (6.2)	0.9 [0.6–1.2]	
**Low fruit and vegetable intake**				0.406			0.303
No	990 (21.3)	80 (8.0)	1		78 (7.9)	1	
Yes	3667 (78.7)	327 (8.9)	1.1 [0.9–1.4]		254 (6.9)	0.9 [0.7–1.1]	
**Low physical activity practice**				0.001			0.484
No	2380 (51.0)	238 (10.0)	1		175 (7.4)	1	
Yes	2284 (49.0)	169 (7.3)	0.7 [0.6–0.9]		156 (6.8)	0.9 [0.7–1.2]	
**Smoking**				0.264			0.156
No	4510 (96.5)	397 (8.8)	1		16 (9.9)	1	
Yes	161 (3.5)	10 (6.2)	0.7 [0.4–1.3]		316 (7.0)	0.9 [0.9–2.5]	

CI: Confidence interval; OR: Odds ratio.

### Prevalence of stroke risk factors

The prevalence of behavioral risk factors was estimated at: 17.2% (95% confidence interval (CI) [16.1–18.3]) for alcohol consumption, 21.5% (95%CI [20.1–22.5]) for low fruit and vegetable intake, 51.1% (95%CI [49.6–52.5]) for low physical activity practice and 3.5% (95%CI [2.9–4.0]) for smoking. Concerning the metabolic risk factors for stroke, the prevalence was estimated at: 8.7% (95%CI [7.9–9.6]) for obesity and 7.1% (95%CI [6.4–7.9]) for high blood pressure. The prevalence of self-reported diabetes and dyslipidemia was respectively estimated at 1.7% (95%CI [1.0–1.6]) and 2.2% (95%CI [1.9–2.7]).

### Factors associated with metabolic risk factors for stroke

Data on univariate analysis are displayed in Tables 1 and 2. All the sociodemographic variables were associated with obesity and the high blood pressure, excepted for the variable sex (p = 0.653) that was not associated with high blood pressure (Table 1).

**Table 2:** Prevalence of diabetes mellitus and dyslipidemia according to socio-demographic and to behavioral characteristics.

	N (%)	Diabetes			Dyslipidemia		
		N (%)	Crude OR [95%CI]	p	N (%)	Crude OR [95%CI]	p
**Sample**	4671 (100)	59 (1.3)			104 (2.2)		
**Age (years)**				<0.001			<0.001
15–29	3198 (68.5)	32 (1.0)	1		67 (2.1)	1	
30–44	927 (19.8)	7 (0.8)	0.8 [0.3–1.7]		18 (1.9)	0.9 [0.6–1.6]	
44–59	362 (7.8)	9 (2.5)	2.5 [1.2–5.3]		11 (3.1)	1.5 [0.8–2.8]	
≥60	184 (3.9)	11 (6.0)	6.2 [3.1–12.7]		8 (4.4)	2.1 [1.0–4.5]	
**Sex**				0.179			0.457
Female	2365 (50.6)	35 (1.5)	1		49 (2.1)	1	
Male	2306 (49.4)	24 (1.0)	0.7 [0.4–1.2]		55 (2.4)	1.2 [0.8–1.7]	
**School education level**				0.721			0.115
None	807 (17.3)	11 (1.4)	1		17 (2.1)	1	
Primary	875 (18.7)	13 (1.5)	1.1 [0.5–2.5]		19 (3.3)	1.6 [0.9–2.9]	
Secondary	2581 (55.3)	32 (1.2)	0.9 [0.5–1.8]		50 (2.0)	0.9 [0.5–1.6]	
Universitary	408 (8.7)	3 (0.7)	0.5 [0.2–1.9]		8 (2.0)	0.9 [0.4–2.2]	
**Marital status**				0.241			0.572
Couple	2022 (43.3)	30 (1.5)	1		48 (2.4)	1	
Alone*	2649 (56.7)	29 (1.1)	0.7 [0.4–1.2]		56 (2.1)	0.9 [0.6–1.3]	
**Professional activity**				0.116			<0.001
Craftsman	1859 (39.8)	19 (1.0)	1		38 (2.1)	1	
Worker/farmer	1320 (28.3)	13 (1.0)	1.0 [0.5–1.9]		27 (2.1)	1.0 [0.6–1.6]	
Resellers	759 (16.3)	12 (1.6)	1.6 [0.8–3.2]		24 (3.2)	1.6 [0.9–2.6]	
No economic activity/others	733 (15.6)	15 (2.1)	2.0 [1.0–4.0]		15 (2.1)	1.0 [0.5–1.8]	
**Alcohol consumption**				0.178			0.756
No	3867 (82.8)	45 (1.2)	1		85 (2.2)	1	
Yes	801 (17.2)	14 (1.8)	1.5 [0.8–2.8]		19 (2.4)	1.1 [0.7–1.8]	
**Low fruit and vegetable intake**				0.716			0.246
No	1004 (21.5)	11 (1.1)	1		28 (2.8)	1	
Yes	3667 (78.5)	46 (1.3)	1.1 [0.6–2.2]		76 (2.1)	0.8 [0.5–1.2]	
**Low physical activity practice**				0.223			0.033
No	2291 (49.0)	33 (1.5)	1		61 (2.7)	1	
Yes	2380 (51.0)	25 (1.1)	1.4 [0.8–2.3]		43 (1.8)	0.7 [0.4–0.9]	
**Smoking**				0.034			0.003
No	4510 (96.5)	54 (1.2)	1		95 (2.1)	1	
Yes	161 (3.5)	5 (3.1)	2.6 [1.1–6.7]		9 (5.6)	2.7 [1.4–5.5]	

CI: Confidence interval; OR: Odds ratio.

In contrary, no behavioral factors were neither associated with obesity nor with high blood pressure, excepted for the low physical activity practice (p = 0.002) that was significantly associated with obesity (Table 1). As for the self-reported diabetes mellitus, only the variables age (p < 0.001) and smoking (p = 0.034) were significantly associated (Table 2). Concerning the dyslipidemia, the association with age (p < 0.001), professional activity (p < 0.001), low physical activity practice (p = 0.035) and smoking (p = 0.003) was significant (Table 2).

Data on multivariate analysis are displayed in Tables 3 and 4. Age (p<0.001), sex (p<0.001), marital status (p<0.001) and professional occupation (p = 0.010) were associated with obesity (Table 3). The obesity was less prevalent in men (adjusted prevalence-ratio (aOR) = 0.4; 95% CI [0.3–3.3]) compared to women and in contrary, more prevalent in older participants compared to those aged 15 to 29 years. It was also more prevalent in participants living in couple (aOR = 2.2; 95% CI [1.6–3.1]) than those living alone. Concerning the professional occupation, the resellers had the highest prevalence (aOR = 3.5; 95% CI [2.1–5.6]) compared to participants who had other occupations.

**Table 3:** Factors associated with obesity and high blood pressure, multivariable analysis, Parakou 2016.

	Obesity		High blood pressure	
	aOR [95% CI]	p	aOR [95% CI]	p
**Age** (years)		<0.001		<0.001
30-44/15-29	2.5 [1.9–3.3]		1.7 [1.2–2.4]	
45-59/15-29	2.4 [1.7–3.4]		4.4 [3.1–6.2]	
≥60/15-29	1.2 [0.7–2.1]		3.5 [2.2–5.4]	
**Sex** (male/female)	0.4 [0.3–0.5]	<0.001	--	--
**Marital status** (couple/alone)	2.2 [1.6–3.1]	<0.001	1.7 [1.4–2.6]	<0.001
**Professional occupation**		0.010		--
Worker or farmer/craftsman	2.7 [1.7–4.4]		--	
Reseller / craftsman	3.5 [2.1–5.6]		--	
No occupation or other /craftsman	1.8 [1.1–3.0]		--	

CI: Confidence interval; aOR: Adjusted odds ratio.

**Table 4:** Factors associated with self-reported diabetes mellitus and dyslipidemia, multivariable analysis, Parakou 2016.

	Diabetes		Dyslipidemia	
	aOR [95% CI]	p	aOR [95% CI]	p
**Age** (years)		<0.001		--
30-44/15-29	0.7 [0.3–1.7]		--	
45-59/15-29	2.4 [1.1–5.1]		--	
≥60/15-29	5.9 [2.9–12.1]		--	
**Low physical activity practice** (yes/no)	--	--	0.7 [0.4–0.9]	0.036
**Smoking** (yes/no)	--	--	2.3 [1.1–4.8]	0.032

CI: Confidence interval; aOR: Adjusted odds ratio.

Only age (p<0.001) and marital status (p<0.001) were significantly associated with high blood pressure after adjustment (Table 3). The prevalence of high blood pressure was higher in older participants compared to “15–29” years old: “30–44” years old (aOR = 1.7; 95% CI [1.2–2.4]), “45–59” years old (aOR = 4.4; 95% CI [3.1–6.2]), and “≥60” years old (aOR = 3.5; 95% CI [2.2–5.4]). This prevalence was also higher in participants living in couple (aOR = 1.7; 95% CI [1.2–2.5]) compared to those living alone.

The prevalence of diabetes increased with the age (Table 4) with the highest prevalence observed among the group of participants aged “≥60” years old (aOR = 5.9; 95% CI [2.9–12.1]). As for dyslipidemia, it was more prevalent among smokers (aOR = 2.3; 95% CI [1.1–4.8]); a lower prevalence was observed among participants who had a low practice physical activity (aOR = 0.7; 95% CI [0.4–0.9]) compared to with those physically active (Table 4).

## Discussion

This study showed the magnitude of stroke risk factors in a sample of relatively young people (mean age: 27.6 ± 12.9 years), in Titirou, in 2016.

The slight female predominance and the high proportion of people with none school education level are in line with national demographic data [10].

The prevalence of alcohol consumption (21.5%) is in the range reported during the STEPS surveys conducted in SSA from 2013 to 2016 (1.4–40.7%) [8]. Smoking (3.5%) was less prevalent compared to STEPS survey results (4.2–13.3%), probably due to the young age of participants. The same observation was made for low fruit and vegetable intake (21.5%) for which the prevalence varied between 67.9–97.6% [8]. Fruit and vegetable intake depends on their availability and accessibility. Our result may be linked to the greater availability of fruits and vegetables in Parakou compared to other towns in Benin. Indeed, Parakou has many gardening areas and is surrounded by fields. On the contrary, low physical activity practice prevalence (51.1%) was higher compared to STEPS survey data (4.3–17.7%) [8].

The prevalence of obesity (8.7%) was in line with the STEPS survey results (1.2–20.5%) while that of high blood pressure (7.1%) was lower. For instance, considering the same definition, 17.6% was reported in Burkina-Faso in 2013, 23.1% in Uganda in 2014, 25.2% in Benin in 2015 [8,9]. Our result could be explained by the fact that the sample comprised younger people while in the STEPS studies, median ages were higher. Concerning the diabetes mellitus and dyslipidemia variables, lower self-reported diabetes mellitus (0.7%) and raised total blood cholesterol (0.4%) were previously noted in the STEPS survey in Benin. Our higher results could be explained by the fact that our study took place in the department of Borgou that has been demonstrated as an area of high prevalence of diabetes [12,13]. Further studies more focused in this area and based on the blood glucose measurement could allow comparisons.

Age and gender were significantly associated with obesity. The results are consistent with the literature data. The increase of obesity with age as observed in our study was previously described [8]. In addition, a positive association between obesity and female gender was noted during several cross-sectional studies in SSA, Brazil, and China [8,14–18]; these results were explained by social and cultural factors. However, a contrary result (higher prevalence of obesity in men than women) was reported by Boua et al. during a study in demographic and health surveillance site in Burkina Faso [19].

The prevalence of obesity was higher among participants living in couples than those living alone and could be explained by the fact that couples might be more sedentary than those living alone. Dagne et al. in Ethiopia had also noted that being married increased the risk of obesity [20]. Resellers seemed more obese than people who practice other activities, probably because of differences in lifestyle.

High blood pressure prevalence increased with the age, but the classic linear association [21–23] was not observed. Contrary to literature data, no association was noted neither between obesity and behavioral factors, nor between high blood pressure and behavioral factors. A larger study could show these associations.

The link between age and diabetes was confirmed in this study. In fact, diabetes increased linearly with age as previously reported in some steps surveys in SSA [8]. As one might expect, a positive association was observed between dyslipidemia and smoking. On the contrary, dyslipidemia was inversely linked to physical activity practice. One explanation to this trend could be that people who knew their status concerning cholesterol rate may be more sensitized to regular physical activity practice than the others.

### Strengths and limitations of the study

This study used a methodology that allowed extrapolating the results to Titirou District. Otherwise, the sample comprised young people aged 15 to 17 years that was not considered in the STEPS surveys. The results provided data on stroke risk factors that could be used for the implementation of targeted interventions in Titirou. For scientific communities, these data showed that hypertension, diabetes, and raised total blood cholesterol are not, yet the main metabolic risk factors in young population in Benin. The main one is rather obesity according to the results obtained in our study, calling then for actions against obesity by tackling physical inactivity. The data can also be used in an advocacy for strengthening of early detection and management of metabolic risk factors in the peripheral health centers of Parakou.

Except for obesity and high blood pressure, stroke risk factors were assessed based on self-reported information which could have introduced information bias due to a wrong or not worthy declaration. In addition, considering the lack of measurements for diabetes and dyslipidemia diagnosis in this study, the prevalence of diabetes and dyslipidemia may have probably been underestimated.

The sampling could be considered by some experts as non-random despite the door-to-door approach in random directions of the neighborhoods. Another limitation is that the population of Titirou is not representative of the population of Parakou what does not allow us to extrapolate our results to the entire population of Parakou.

## Conclusion

The study revealed that a significant part of the residents of Titirou, aged at least 15 years, lived with stroke risk factors. For primary prevention of stroke in this community, adapted actions, including the physical activity promotion should be implemented. The actions should take into account socio-cultural realities.

## Supporting information

S1 Data Database on the prevalence of stroke risk factors and associated factors in Titirou, Parakou, 2016. Variables: id number, age, sex, level of school education, profession, marital status, weight, height, systolic blood pressure, diastolic blood pressure, low physical activity practice (yes = 1, no = 2), Low fruit and vegetable intake (yes = 1, no = 2), smoking (yes = 1, no = 2), alcohol consumption (yes = 1, no = 2), history of high total blood cholesterol (yes = 1, no = 2), history of diabetes (yes = 1, no = 2).
(XLSX)
